# Histological and Micro-CT Evidence of Stigmatic Rostellum Receptivity Promoting Auto-Pollination in the Madagascan Orchid *Bulbophyllum* *bicoloratum*

**DOI:** 10.1371/journal.pone.0072688

**Published:** 2013-08-13

**Authors:** Alexander Gamisch, Yannick M. Staedler, Jürg Schönenberger, Gunter A. Fischer, Hans Peter Comes

**Affiliations:** 1 Department of Organismic Biology, University of Salzburg, Salzburg, Austria; 2 Department of Structural and Functional Botany, University of Vienna, Vienna, Austria; 3 Flora Conservation Department, Kadoorie Farm and Botanic Garden Corporation, Hong Kong, China; University of Nottingham, United Kingdom

## Abstract

**Background:**

The rostellum, a projecting part of the gynostemium in orchid flowers, separates the anther(s) from the stigma and thus commonly prevents auto-pollination. Nonetheless, as a modified (usually distal) portion of the median stigma lobe, the rostellum has been frequently invoked of having re-gained a stigmatic function in rare cases of orchid auto-pollination. Here it is shown that a newly discovered selfing variant of Madagascan *Bulbophyllum* *bicoloratum* has evolved a modified rostellum allowing the penetration of pollen tubes from *in situ* pollinia.

**Methods:**

Gynostemium micro-morphology and anatomy of selfing and outcrossing variants of *B* *. bicoloratum* was studied by using light and scanning electron microscopy and histological sections. Pollen tube growth in the selfing variant was further observed via X-ray computed microtomography (micro-CT), providing 3D reconstructions of floral tissues at a micron scale.

**Findings:**

Selfing variants possess a suberect (‘displaced’) rostellum rather than the conventional, erect type. Very early in anthesis, the pollinia of selfers are released from the anther and slide down onto the suberect rostellum, where pollen tube growth preferentially occurs through the non-vascularized, i.e. rear (adaxial) and (semi-) lateral parts. This penetrated tissue is comprised of a thin layer of elongate and loosely arranged cells, embedded in stigmatic exudates, as also observed in the stigmatic cavity of both selfing and outcrossing variants.

**Conclusions:**

Our results provide the first solid evidence of a stigmatic function for the rostellum in orchid flowers, thereby demonstrating for the first time the feasibility of the micro-CT technique for accurately visualizing pollen tube growth in flowering plants. Rostellum receptivity in *B* *. bicoloratum* probably uniquely evolved as an adaptation for reproductive assurance from an outcrossing ancestor possessing an erect (non-receptive) rostellum. These findings open up new avenues in the investigation of an organ that apparently re-gained its ‘primordial function’ of being penetrated by pollen tubes.

## Introduction

Ever since Darwin [1,2], orchids are universally acknowledged to rank amongst the most singular and modified organisms in the plant kingdom. One of the most extraordinary and unique organs of Orchidaceae, the rostellum, has long attracted the attention of botanists and evolutionary biologists [3,4]. When defined by its function, the rostellum is foremost a versatile physical barrier between the anther(s) and the stigma of the same flower, and is generally regarded unsuitable for pollen germination, thus preventing autonomous self-pollination ([4,5,6] but see below). In addition, as the rostellum produces viscous fluid or viscous appendages, its second main function is to anchor the pollinia firmly on the pollinating vector [1,2,4,5,7]. However, the origin and nature of the rostellum is still the subject of considerable debate (for reviews, see 5,6,8,9).

From Darwin [1,2] onwards, the rostellum was widely believed to be a modified structure of the entire median (‘third’) stigma lobe having become non-receptive (sterile) and impenetrable to pollen tubes. In fact, some authors proposed using the term ‘rostellum’ in a broad sense including the entire median stigma lobe, with the modified extension then termed ‘rostellar projection’ [10–12]. However, ontogenetic studies in all monandrous orchids investigated so far (e.g. Orchidoideae, Spiranthoideae, Epidendroideae) revealed that this modified extension develops directly from only the distal portion of the median stigma lobe, while the proximal (and usually smaller) portion, together with the two lateral stigma lobes, forms the receptive stigmatic surface [13–15]. Hence, the interpretation of the rostellum as a modified (usually distal) portion of the median stigma lobe is now accepted almost generally [14,16]. That said, in some Orchideae the ‘rostellum’ still might have different origins, possibly being derived from, e.g., the lateral stigma lobes [17], staminodes ( *Herminium* *monorchis* , *Coeloglossum* *viride* [18]:) or the fertile anther ( *Stereosandra* [19]:).

Orchids have evolved a variety of autonomous self-pollination mechanisms (reviewed in 3). In most of these cases, the rostellum either does not develop, or more rarely, develops incompletely or secondarily disintegrates (e.g. *Calanthe* *mannii* [20]:), or bears stigmatic papillae along the whole lower front up to the apex (e.g. *Cephalanthera* *rubra* *,* *Epipactis* *helleborine* *,* *E* *. purpurea* *,* *Habenaria* *hyperborea* , *Spiranthes* *spiralis* [18]:), facilitating contact between (in all those examples) friable pollinia and stigmatic fluid [18,20]. In other rare instances of orchid self-pollination, individual grains, tetrads, or granular masses of pollen (massulae) fall directly onto each of three receptive lobes situated below. This type of autogamy has been observed in *Platanthera* *clavellata* [21] and *Cyrtorchis* *aphylla* [22]. Catling [3,21] interpreted the median lobe of the former species as being homologous to the rostellum. However, there is no firm evidence yet (e.g. ontogenetic, morphological, phylogenetic) in support of such a homology, and the alternative hypothesis of a complete lack of the rostellum cannot be excluded (see 20,22). In fact, we are aware of only a single study supporting the oft-stated view (e.g. [2,3,5,18,23,24]) that the rostellum itself may function as a stigma. Thus, in *Eulophia* *nyasae* , a terrestrial orchid from South-Central Africa, pollen tubes growing from *in situ* pollinia were documented to penetrate a morphologically distinct rostellum on their way down to the stigma [25]. However, in the latter study no evidence was recorded of fruit set from supposedly auto-pollinated flowers, and neither in this or any other orchid species has this process of ‘rostellum penetration’ been demonstrated yet by more detailed micro-morphological and histological evidence.

**Figure 1:**
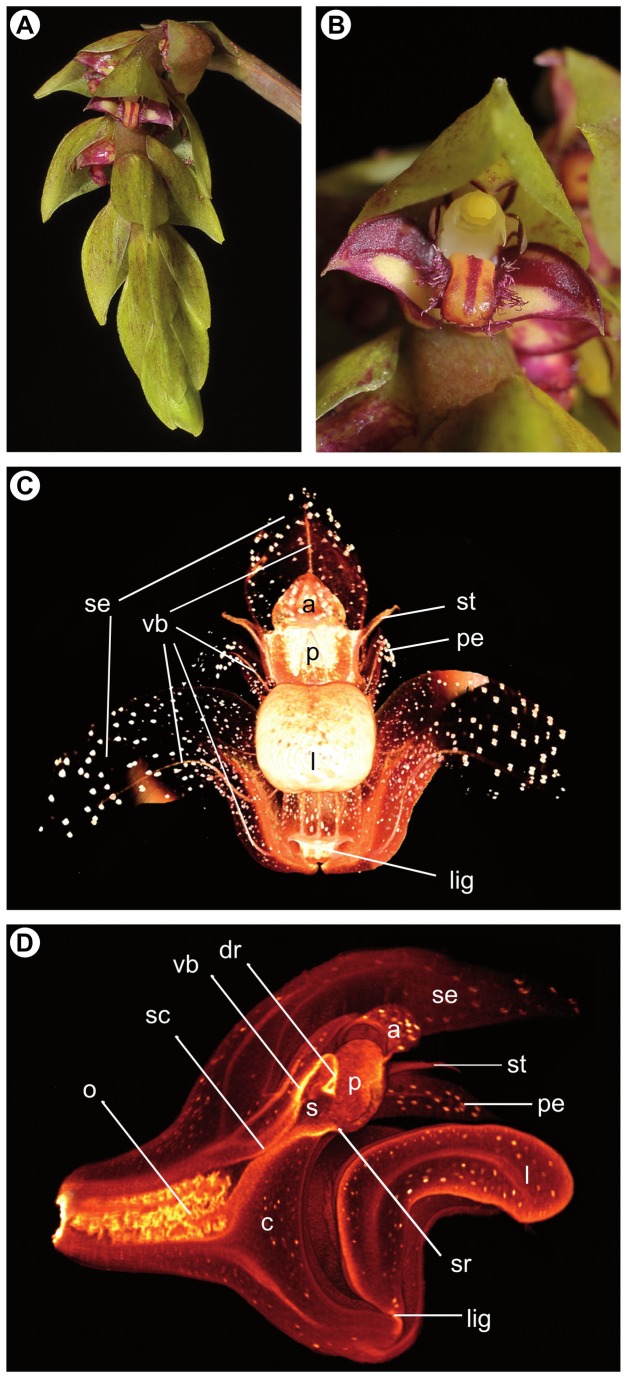
Floral features of *Bulbophyllum bicoloratum*. (A) Inflorescence with spirally arranged, resupinate flowers; (B) flower (*ca*. 4 x 6 mm) covered by an imbricate, oval-acute bract; (C) virtual 3D model generated by micro-CT of an auto-pollinated *B*. *bicoloratum* flower; (D) longitudinal section of the same 3D model passing through the median plane of the flower (see also Videos S1 and S2 corresponding to Figure 1C and D, respectively). In the 3D models, idioblasts with dense raphide-bundles are visible as white dots scattered throughout the non-reproductive floral tissue. Abbreviations: a, anther; c, column (gynostemium); dr, ‘displaced’ (suberect) rostellum; l, labellum; lig, ligament (hinge); o, ovary; p, pollinia; pe, petalum; s, stigmatic cavity; sc, stylar canal; se, sepalum; st, stelidium; sr, rim of stigmatic cavity; vb, vascular bundle. Photo credit Figure A, B: Rogier van Vugt (Leiden Botanical Garden).

*Bulbophyllum* *bicoloratum* Schltr. (Epidendroideae) is an epiphytic orchid endemic to Madagascar, where it has been recorded from northwestern low to mid altitude (400–800 m) evergreen forests, southeastern littoral forests (0–70 m), and isolated pockets of marshland and gallery forests of the drier Central Plateau (800–1300 m) ( [26,27] Fischer et al., unpubl. Data). This species is characterized by small, ovoid pseudobulbs, oblong leaves, and one or few many-flowered inflorescences with small (*ca* 4 x 6 mm), resupinate, pale green to reddish flowers, arranged in three rows and completely covered by imbricate, oval-acute bracts (Figure 1A, B). The ciliated labellum (‘lip’) is tongue-shaped, thick, fleshy and motile, elastically hinged at the base of the up-curved gynostemium (column), which is formed by the union of androecium and gynoecium (Figure 1B–D, and Video S1, available online). In this species, the single anther is usually separated from the deeply concave stigma below by an erect rostellum, as is typical for the predominantly outcrossing and fly- (or more rarely bee-) pollinated species of this mostly self-compatible genus [5,28–30]. However, as part of ongoing phylogenetic and mating system studies in Madagascan *Bulbophyllum* (Gamisch et al., unpubl. data), we have found that some individuals of *B* *. bicoloratum* produce only flowers, in which the rostellum is suberect or ‘displaced’ (see below). Moreover, when grown under greenhouse conditions and subjected to experiments of bagged self-pollination, these plants proved capable of autonomous, profuse fruit set for about 90% of their flowers; by contrast, individuals with the usual erect rostellum never showed any autonomous fruit set, indicative of a predominantly outcrossing mode of reproduction (Gamisch et al., unpubl. data). These observations not only suggest that *B* *. bicoloratum* is divided into selfing and outcrossing variants differing structurally from each other, but also that a highly efficient auto-pollination mechanism exists in selfing variants of this species despite the presence of a well-developed rostellum.

The main aim of the present paper is to describe this auto-pollination process in selfing variants of *B* *. bicoloratum* in greater detail using conventional microscopic and histological methods in combination with a newly developed staining protocol (Staedler et al., unpubl. data) for X-ray micro-computed tomography (micro-CT), capable of providing 3D-reconstructions of plant tissues at the micron scale (1.5 µm resolution) with no damage of surrounding structures [31,32]. Thus not only was it our intent to examine the function of the rostellum in *B* *. bicoloratum* auto-pollination in its own right, and hence to begin to assess the generality of earlier suggestions and preliminary findings concerning rostellum stigmatic receptivity in orchids [3,5,25], but also to assess for the first time the utility of micro-CT for studying pollen tube growth in flowering plants.

## Materials and Methods

### Plant material

Flowers just before anthesis and open flowers were harvested from both selfing and outcrossing plants of *B* *. bicoloratum* cultivated under greenhouse conditions at the Botanical Garden of Salzburg University (HBS). All plants were collected with permits issued by the Département des Eaux et Fôrets (as part of a 10 years ongoing collaboration between the Parc Botanique et Zoologique de Tsimbazaza, the University of Vienna and the University of Salzburg) from one population each in the northwestern Malagasy provinces of Mahajanga, and Antsiranana and the southeastern province of Fianarantsoa (HBS collection numbers FS5686, FS5687, FS5695, FS5696, FS5697, FS5832, FS5849, FS5852, FS1052, FS807, FS5710, FS5709, FS5684).

### Ethics Statement

All necessary permits were obtained for the described study, which complied with all relevant regulations.

### Microscopical, micro-CT and histological methods

To examine the general morphological structure of the gynostemium (column), 26 fresh flowers of 13 individual plants were dissected, observed, and photographed using a Leica EZ4D stereomicroscope (Leica Microsystems, Wetzlar, Germany). For scanning electron microscopy (SEM), three flowers of one selfing individual were preserved in standard formalin-aceto-alcohol (FAA; absolute ethanol, 90%; glacial acetic acid, 5%; formalin, 5%). Dissected columns of this fixed material were washed, dehydrated through a graded ethanol series, and dried in a Bal-Tec CPD 030 critical point dryer (Bal-Tec AG, Balzers, Liechtenstein). The samples were then mounted on aluminium stubs with colloidal carbon, coated with gold using a sputter coater (Agar Scientific, Essex, UK) for 90 s, and observed under a Philips XL-30 ESEM scanning electron microscope (FEI Electron Optics, Eindhoven, The Netherlands) operated at 10–15 kV.

For micro-CT observations of overall flower structure, vascular bundle arrangement and pollen tube growth, three flowers of one selfing individual were temporarily transferred into ‘Copenhagen Mix’ (absolute alcohol, 70%; glycerol, 2%; water, 28%) and then infiltrated with 1% phosphotungtic acid (PTA) in 70% ethanol for seven days in order to increase contrast [33]. The contrasting solution was changed on a daily basis. The samples were mounted inside 100-µL pipette tips, submerged in infiltration medium to prevent desiccation during scanning. The scans were performed on a MicroXCT-200 imaging system (Xradia, Pleasanton, CA, USA) with a L9421-02 90kV Microfocus X-ray (MFX) source (Hamamatsu Photonics, Iwata City, Japan). Scans were performed using the following settings: acceleration voltage, 50 kV; source current, 100 µA; exposure time, 30 s; pictures per sample, 1200; camera binning, 1; optical magnification, 4 x and 20 x, with pixel sizes of 2.4 µm and 0.44 µm, respectively. The total exposure time was approximately 10 hours for each sample. Animated 3D images of these micro-CT scans are provided as Supplementary Videos (available online; see below).

For microtome thin sectioning, four flowers of one selfing individual and two flowers of one outcrossing individual were ﬁxed in an aqueous solution of FAA (absolute ethanol, 50%; glacial acetic acid, 10%; formalin, 5%; water, 35%) and stored therein or in 70% ethanol. Dissected columns of this material were embedded in 2-hydroxyethyl methacrylate (Kulzer’s Technovit 7100; Heraeus Kulzer, Wehrheim, Germany), cut using a Microm HM rotary microtome 355S (Microm, Walldorf, Germany) at 10 µm, and stained with ruthenium red and toluidine blue [34]. Ruthenium red binds to pectins and acid mucopolysaccharides [35] and is commonly used to stain pectinaceous material and carbohydrates in stigmatic exudates [36,37]. Toluidine blue is an acidophilic metachromatic dye that selectively stains acidic tissue components and lignin [38,39], and is used as a counterstain to ruthenium red to improve image resolution and contrast [40]. Permanent slides are deposited at the Department of Structural and Functional Botany, University of Vienna.

## Results

### Gynostemium micro-morphology of outcrossers and selfers

**Figure 2:**
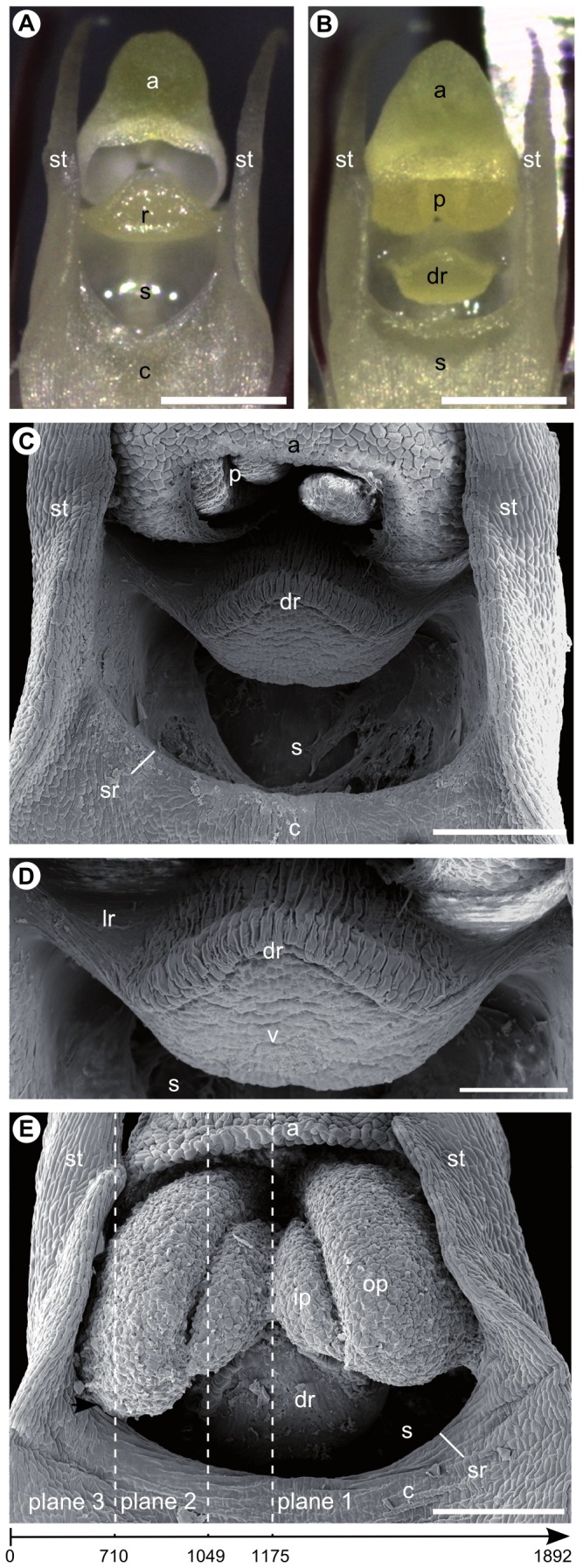
Apical gynostemium structures of *Bulbophyllum bicoloratum* viewed under the stereomicroscope (A, B) and scanning electron microscope (C–E). (A) Isolated column of an outcrossing individual with erect rostellum at anthesis (without pollinia); (B) isolated column of a selfing individual with ‘displaced’ (suberect) rostellum at pre-anthesis (note, at anthesis, swollen pollina will obstruct the view on the rostellum; compare E); (C) column of a selfing individual with pollinia still in the anther at pre-anthesis; (D) close-up of the ‘displaced’ (suberect) rostellum (same as C) with the viscidium at its apex and cuticular folds on the upper (adaxial) side (except for the lateral sides); (E) column of a selfing individual after pollinia have been released from the anther onto the ‘displaced’ (suberect) rostellum. The swollen outer pollinia contact the (semi-) lateral rims of the stigmatic cavity (arrow). The dashed lines of planes 1–3 indicate approximate positions of longitudinal micro-CT sections shown in Figure 4A–C, respectively. The arrow below indicates the directionality of the complete scan (see Video S3), with numbers identifying scan-frames roughly corresponding to planes 1–3. Abbreviations: a, anther; c, column (gynostemium); dr, ‘displaced’ (suberect) rostellum (with cuticular folding); lr, lateral sides of ‘displaced’ (suberect) rostellum (devoid of cuticular folding); p, pollinia; ip, inner pollinium; op, outer pollinium; r, rostellum; s, stigmatic cavity; sr, rim of stigmatic cavity; st, stelidium; v, viscidium. Scale bars: (A, B) = 0.5 mm; (C, E) = 0.2 mm; (D) = 0.1 mm.

**Figure 3:**
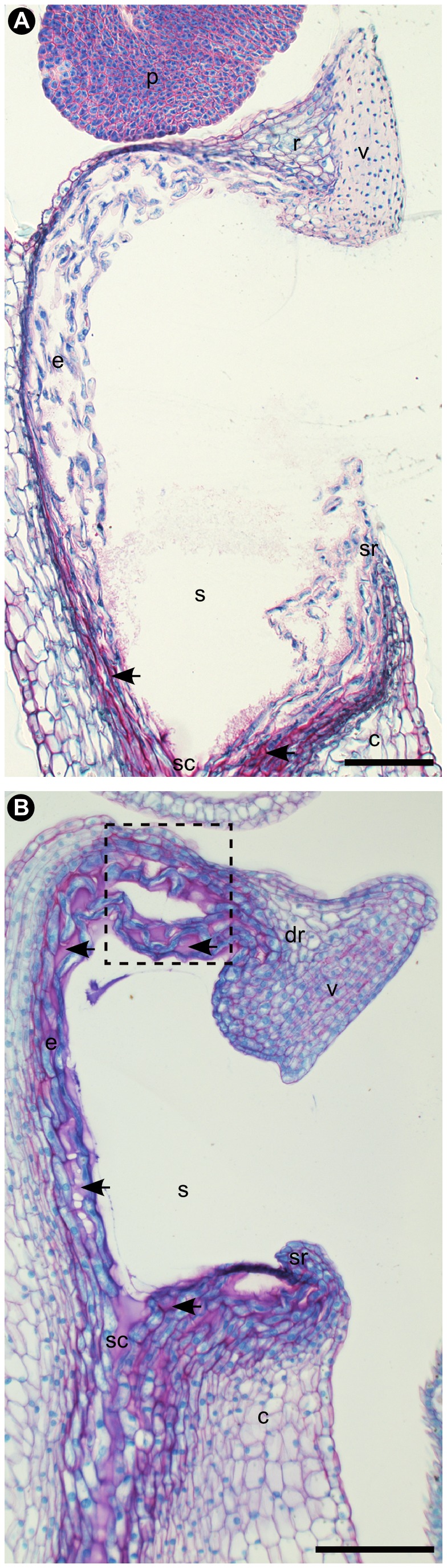
Longitudinal semithin sections through the stigmatic cavity and rostellum of an outcrosser (A) and selfer (B) of *Bulbophyllum bicoloratum.* Sections were taken between the center and periphery of the rostellum, and stained with ruthenium red/toluidine blue. In (A), red stained mucilage is visible only at the base of the stigmatic cavity and the adjacent stylar canal region (arrows), while in (B) it covers the entire stigmatic cavity (arrows) and extends throughout the non-vascularized, (semi-) lateral and rear (adaxial) parts of the rostellum (black rectangle), where pollen tube growth apparently takes place (see Figure 4B, F, G). Abbreviations: c, column; dr, ‘displaced’ (suberect) rostellum; e, elongate and loosely arranged cells of the stigma and (adaxial) parts of the rostellum; p, pollinium; r, rostellum; s, stigmatic cavity; sc, stylar canal; sr, rim of stigmatic cavity; v, viscidium. Scale bars: (A, B) = 0.1 mm.

**Figure 4:**
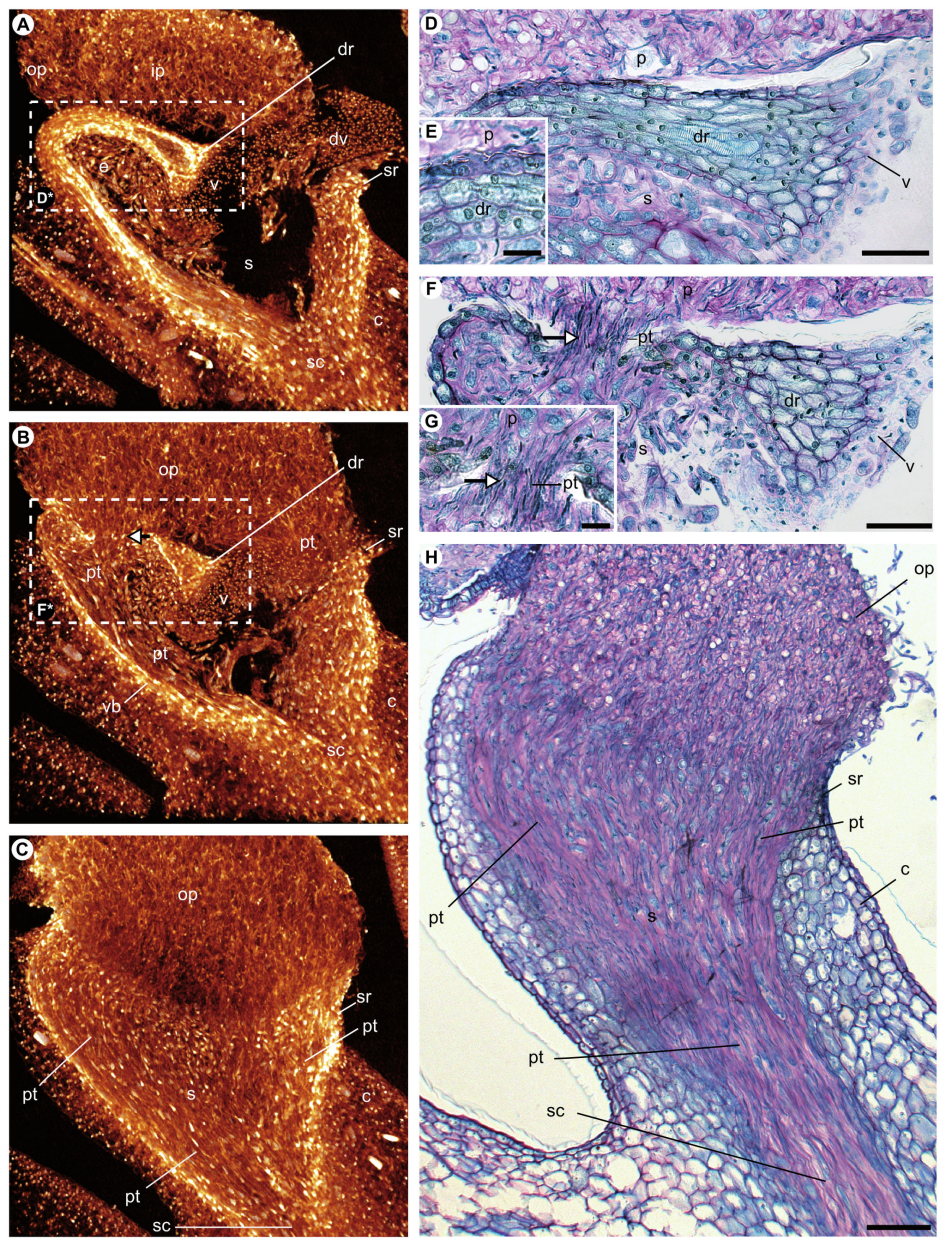
Longitudinal sections through the gynostemium apex of an auto-pollinated flower of *Bulbophyllum bicoloratum*. Virtual micro-CT sections (slice thickness = 66.5 µm) were taken along the central (A), ‘intermediate’ (B), and peripheral (C) planes, approximately corresponding to the dashed lines of planes 1–3 in Figure 2E (see also corresponding scan-frames 1175/1892, 1049/1892 and 710/1892 in Video S3). (D–H) Corresponding longitudinal semithin sections through the rostellum along its (D) near-central plane 1 (detailed in E), (F) ‘intermediate’ plane 2 (detailed in G), and (H) peripheral plane 3, and stained with ruthenium red/toluidine blue. The white dashed rectangles [D*] and [F*] in (A) and (B) indicate the approximate positions of the histological sections depicted in (D) and (F), respectively. Note pollen tubes growing through both the non-vascularized, (semi-) lateral parts of the rostellum (see arrows in B, F and G) and the (semi-) lateral rims of the stigmatic cavity (B, C and H). See text for further details. Abbreviations: c, column; dr, ‘displaced’ (suberect) rostellum; dv, detached viscidium tissue (possibly artifact); e, elongate and loosely arranged cells of the stigma; ip, inner pollinium; op, outer pollinium; p, pollinium; pt, pollen tubes; s, stigmatic cavity; sc, stylar canal; sr, rim of stigmatic cavity; v, viscidium; vb, vascular bundle. Scale bars: (D, F, H) = 0.05 mm; (E, G) = 0.01 mm.

No gross morphological differences in vegetative or floral phenotype were observed between outcrossing and selfing individuals of *B* *. bicoloratum* . Likewise, apart from subtle differences in the relative position, structure, and function of their rostellum (see below), both mating types were found to share essentially identical characteristics in overall gynostemium micro-morphology. These can be summarized as follows: (1) the gynostemium is ‘winged’, with elongated and slender column arms or ‘stelidia’ on each side, which are usually interpreted as staminodia [41]; (2) it terminates into a single, two-chambered anther with four, hard, non-friable pollinia, in two pairs, unequal in size, and without appendages (Figures 1C, D and 2); (3) the anther/pollinia are spatially separated from the deeply concave stigma below by a distinctly protruding rostellum (Figure 2A, B); (4) the anterior part of the rostellum is comprised of a single, fleshy and sticky pad-like structure or ‘viscidium’ (Figure 2C, D), as it is typical for the genus [30]; (5) at anthesis, the stigmatic cavity is filled by a jelly-like, viscous fluid, as also visible macroscopically; and (6) both the stigmatic cavity and the central part of the rostellum are supplied by massive vascular bundles, which on their way down to the pedicel are narrowing towards the stylar canal before spreading out again at the ovary (Figure 1D). Notably, the distal part of the single, unbranched vascular bundle traversing the central part of the rostellum forms a funnel-shaped structure (see also Video S2).

As to the major structural difference between the two mating types, the rostellum of outcrossers is erect so that a spatial separation between anther/pollinia and rostellum is almost absent (Figure 2A). In selfers, in contrast, the distance between pollinia and rostellum is slightly increased due to a lowered, i.e. suberect or ‘displaced’ position of the rostellum part down towards the stigmatic cavity (Figure 2B, C). Observations on intact flowers showed that the pollinia of selfers are released from the anther very early in anthesis, and slide down onto the suberect rostellum; at a slightly later stage, parts of the two outer and larger pollinia, after having swollen, usually come into contact with the (semi-) lateral rims of the stigmatic cavity (see Figure 2E).

### Histological analyses of the stigma and rostellum in outcrossers and selfers

Histological semithin sections indicate that the stigmatic cavity of both outcrossing and selfing individuals of *B* *. bicoloratum* is lacking a distinct cuticular layer deposited on the outer surfaces of the epidermal cells (Figure 3A, B), which thus accords with similar reports in other epidendroid orchids [42]. Rather, in both mating types, the entire stigmatic cavity is padded with several layers of non-papillate, elongate and loosely arranged cells, forming a continuous stigmatic surface extending from approximately the apical portion of the lower (abaxial) surface of the rostellum (excluding the viscidium) down to the stylar canal (Figure 3A, B). In outcrossers, only those elongate and loosely arranged cells located at the base of the stigmatic cavity and in the adjacent stylar canal region were found to be embedded in an extra-cellular mucilage that strongly stains with ruthenium red (Figure 3A), indicating the presence of stigmatic exudates there. By contrast, in selfers, all these cells are generally embedded in a relatively thick layer of this red-stained mucilage, which thus not only covers the entire stigmatic cavity and the wall of the stylar canal but also extends throughout the non-vascularized, rear (adaxial) and (semi-) lateral parts of the rostellum (see black rectangle in Figure 3B, and below).

### Micro-CT and histological observations on pollen tube growth in selfers

In order to study pollen tube growth in selfers, longitudinal micro-CT sections of an auto-pollinated flower were visualized from the near-centre towards the periphery of the apical portion of the gynostemium (Figure 4A–C and Video S3), with their approximate positions indicated in Figure 2E (see dashed lines of planes 1–3). Considering the near-central section (Figure 4A; corresponding to plane 1 in Figure 2E), no pollen tubes were observed penetrating the rostellum along its median plane (vascular bundle); pollen tubes were also not observed penetrating the distal rim of the stigmatic cavity, probably due to its lack of contact with the pollinia (see also Figure 2E). Corresponding histological semithin sections likewise provided no evidence for any pollen tube growth in the central part of the rostellum, where the vascular bundle has its largest extent (Figure 4D, E). However, along the ‘intermediate’ plane (Figure 4B; corresponding to plane 2 in Figure 2E), pollen tubes were observed not only growing from the outer pollinia into the semi-lateral rim of the stigmatic cavity but also penetrating the rear (adaxial) and semi-lateral parts of the rostellum, where vascular tissue is lacking. Histological analyses further indicate that these latter areas of the rostellum comprise a thin layer of elongate and loosely arranged cells, embedded in intensely ruthenium red-stained mucilage (Figure 4F, G; see also black rectangle in Figure 3B). In the peripheral plane (Figure 4C, H; corresponding to plane 3 in Figure 2E), pollen tubes of the outer pollinia were observed to grow unhindered through the lateral parts of the rostellum and the lateral rim of the stigmatic cavity until reaching the ovary. Taken together, these observations are indicative of the presence of stigmatic tissue in the non-vascularized, i.e. adaxial and (semi-) lateral parts of the rostellum, allowing its penetration by pollen tubes.

## Discussion

Our results show that the suberect rostellum of auto-pollinating (‘selfing’) variants of *B* *. bicoloratum* has a stigmatic function by allowing the penetration of pollen tubes from *in situ* pollinia, as evidenced by both histological sections and micro-CT scans (Figure 4 and Video S3). These observations confirm numerous earlier predictions of rostellum receptivity as a mechanism promoting auto-pollination in orchids (e.g. [2,3,5,18,23–25]). However, firm evidence in support of this notion has so far been exceedingly scarce and, to the best of our knowledge, restricted to the descriptions and drawings of Williamson [25], who raised the possibility that ‘the central part of the rostellum [in *E* *. nyassae* ] contains some stigmatic tissue’. Instead, for selfing *B* *. bicoloratum* , the present findings clearly demonstrate that pollen tube growth into the rostellum occurs preferentially through its non-vascularized, i.e. rear (adaxial) and (semi-) lateral rather than central parts. That said, auto-pollination in this variant is potentially not entirely dependent on the receptivity of the rostellum, as we also observed pollen tube growth from the outer pollinia into the (semi-) lateral rims of the stigmatic cavity (Figure 2E). Apparently, this ‘secondary’ mechanism of auto-pollination is facilitated by the suberect position of the rostellum in combination with the swelling of pollinia due to hydration and vacuolization (see also 43). Also, we may not entirely exclude the possibility of pollinator-mediated outcrossing in this variant, because it still retains a viscidium that usually serves to attach the pollinia to the body of the pollinator, and is therefore often considered typical for outcrossing (e.g. [44]). However, in cultivated individuals, we observed that the pollinia slide down onto the suberect rostellum very early in anthesis. If this holds true in nature, pollinia would be hardly available for siring offspring on other individuals. In lieu of observations of pollinators under natural conditions, outcrossing rates determined by genetic analyses are currently underway to help solving this issue.

Our histological observations of the stigmata in both outcrossing and selfing variants of *B* *. bicoloratum* (Figure 3) provided interesting clues for the interpretation of the micro-CT analyses of rostellum receptivity. Earlier reviews had suggested that, in general, Orchidaceae possess a wet-type stigma with a receptive surface having small- to medium-sized papillae [45,46]. However, subsequent observations on the stigmata of some epidendroid orchids (e.g. *Dendrobium* , *Epidendrum*, Oncidinae) revealed that loosely arranged cells are suspended in the viscous fluid filling the stigma, and this stigma type has been classified as ‘wet-detached-cellular’ [36,42,47,48]. It is clear from the evidence provided herein that the stigma of *B* *. bicoloratum* falls into this latter category, as a large number of such cells were observed within the stigmatic extra-cellular matrix of this species (Figure 3). This mucilage, as shown previously in *Dendrobium* , is rich in carbohydrates and arabinogalactan-proteins secreted from these elongate cells [37]. These cells contain numerous amyloplasts, which are depleted from their storage of starch once pollen tubes have passed through, indicating that they may provide the latter with energy [49]. The arabinogalactan-proteins, in turn, are thought to act as a carbon source for pollen tube growth [37]. As *Bulbophyllum* pollen is known to germinate in 10% saccharose solution [50], and as the stigmatic extra-cellular matrix of *B* *. bicoloratum* stains positively with ruthenium red in a very similar way to that of *Dendrobium* [37], we have little doubt that this mucilage represents a stigmatic exudate functioning as pollen tube transmitting tract. In outcrossing *B* *. bicoloratum* , this exudate is restricted to the base of the stigmatic cavity (Figure 3A), while in the selfing variant it not only covers the entire cavity but also extends, intriguingly, throughout the non-vascularized parts of the rostellum (Figure 3B), where pollen tube penetration has been observed (see Figure 4 and Video S3).

A potential caveat is that we did not demonstrate that pollen penetrating the rostellum of selfing *B* *. bicoloratum* actually fertilizes ovules. However, we observed that cultivated individuals exhibit high levels of fruit set (capsules) from experimentally bagged flowers where auto-pollination took place (Gamisch et al., unpubl. data). Moreover, these self-fertilized capsules contained an abundance of seeds, which proved viable based on enzyme activity as indicated by the tetrazolium test (Jaros, unpubl. data). Even though all these observations have to be treated with caution due to limited sample sizes, we believe this is preliminary circumstantial evidence suggesting that, in selfing *B* *. bicoloratum* , the stigmatic function of the suberect rostellum enables the production of viable seeds.

Still, one may conjecture that this type of rostellum viz. auto-pollination is a mere ‘aberrant’ phenomenon of a rare mutant. This, however, would seem unlikely due to the fact that nine out of 13 *B* *. bicoloratum* plants in our living collection at HBS are comprised of this variant, originating from two geographically distant (*ca.* 130 km) natural populations in Northwest Madagascar (coordinates available upon request), with one of those sites also harbouring the conventional variant. Natural fruit set surveyed in six populations from across the species’ range, and expressed as the proportion of ripe capsules relative to the total number of flowers per inflorescence, approximates 46% (*n* = 78; Jaros, unpubl. data). This is more than 2.5 times the median value reported for non-autogamous orchids from tropical regions (*ca.* 17%, estimated over 91 species [51]). Thus, despite limited sampling, these preliminary data suggest that the specific mode of auto-pollination observed in *B* *. bicoloratum* is probably not ‘aberrant’ but rather a common, if not prevalent mode of reproduction in this species. It has often been stated that selfing is an adaptive, reproductive assurance strategy to cope with specific environmental conditions such as low availability of conspecific potential mates and/or pollinators as a consequence of habitat fragmentation [3,25,52–54]. This could well be the case in *B* *. bicoloratum* , given the severe human-mediated deforestation and degradation that impacted the island’s primary forest and marshland vegetation over the last 1800 years, and especially since the 1950/1970s [55–57]. That said, alternative (e.g. palaeo-climatic) catalysts initiating the evolution of selfing in *B* *. bicoloratum* (and other Madagascan orchids; see below) should not be dismissed so readily, but this is still a hypothesis to be tested against landscape genetic data and (palaeo-climatic) niche modelling approaches tailored to specific ecological requirements of the species of interest [58,59].

A final question of particular relevance concerns the broader evolutionary pathway leading to the origin of a stigmatic rostellum in *B* *. bicoloratum* . Molecular phylogenetic studies in Madagascan *Bulbophyllum* indicate that this species nests firmly within a clade of *ca.* 30 species comprising sects. *Calamaria* , *Humblotiorchis*, and *Bifalcula*, but none of those relatives has a suberect rostellum ( [60] Gamisch et al., unpubl. data). Instead, the conventional erect (non-receptive) rostellum, and thus a potentially outcrossing mating type, appears to be widespread across these sections, even though there is evidence that auto-pollination has evolved independently within a few other species, but exclusively due to the complete abortion of the rostellum (Gamisch et al., unpubl. data). Although further research is required, all available data suggest that auto-pollination via rostellum receptivity in *B* *. bicoloratum* represents a uniquely derived character state that most likely evolved from an outcrossing ancestor possessing an erect (non-receptive) rostellum. Despite capitalizing on a pre-exiting trait, this evolutionary change may have required multiple steps, including shifts in the position, structure, and function of the rostellum, which may also explain its apparent rarity compared with orchid auto-pollination linked to a poorly developed or aborted rostellum [3,25,61–63]. However, taking into account the interpretation of the rostellum as a modified (usually distal) portion of the median stigma lobe [14,16], rostellum receptivity in *B* *. bicoloratum* can also be considered an evolutionary reversal rather than a novelty.

In conclusion, this is the first report providing hard, i.e. histological and micro-CT evidence of auto-pollination in orchids by virtue of a rostellum that may have regained its stigmatic function as part of the distal median stigmatic lobe. While this mode of auto-pollination has been previously documented with drawings in *Eulophia* *nyasae* [25], our study of *Bulbophyllum* *bicoloratum* adds greater detail to this process by visualizing pollen tube growth through the non-vascularized tissues of the rostellum. The 3D micro-CT scans were of great importance in the spatial evaluation of this process and proved invaluable in characterizing the vascular properties of the gynostemium and the flower as a whole. Clearly, further research is needed to reconcile these observations with physiological and, ideally, differential gene expression analyses [64] to gain a better understanding of the biochemical and molecular mechanisms operating during pollen-rostellum interaction in selfing *B* *. bicoloratum* , and in comparison to pollen tube growth occurring in the conventional stigma of outcrossing conspecifics. Similarly, it would be extremely interesting to illuminate the developmental-genetic pathway for this type of rostellum in search for functional genes (e.g. restorers, enhancers) preserved over evolutionary time scales to allow for its ‘primordial function of being penetrated by pollen-tubes’ (cf. [24,65–67]). Overall, we expect that this apparently rare but potentially adaptive mode of auto-pollination will be detected more frequently as additional data are assembled from the world’s most diverse family of flowering plants, whereby micro-CT should offer an efficient use in the noninvasive study of this phenomenon.

## Supporting Information

Video S1 **Rotating, virtual 3D model of the whole flower.**
(see Figure 1C).
(MP4)

Video S2 **Longitudinal cross-section of the 3D model passing through the median plane.**
(see Figure 1D).
(MPG)

Video S3 **Complete, longitudinal micro-CT scan through the gynostemium apex.**
See frames 1175/1892, 1049/1892 and 710/1892 for approximate positions of, respectively, the central (1), ‘intermediate’ (2), and peripheral (3) planes as indicated in Figure 2E and shown in Figure 4A–C, respectively.
(MP4)
