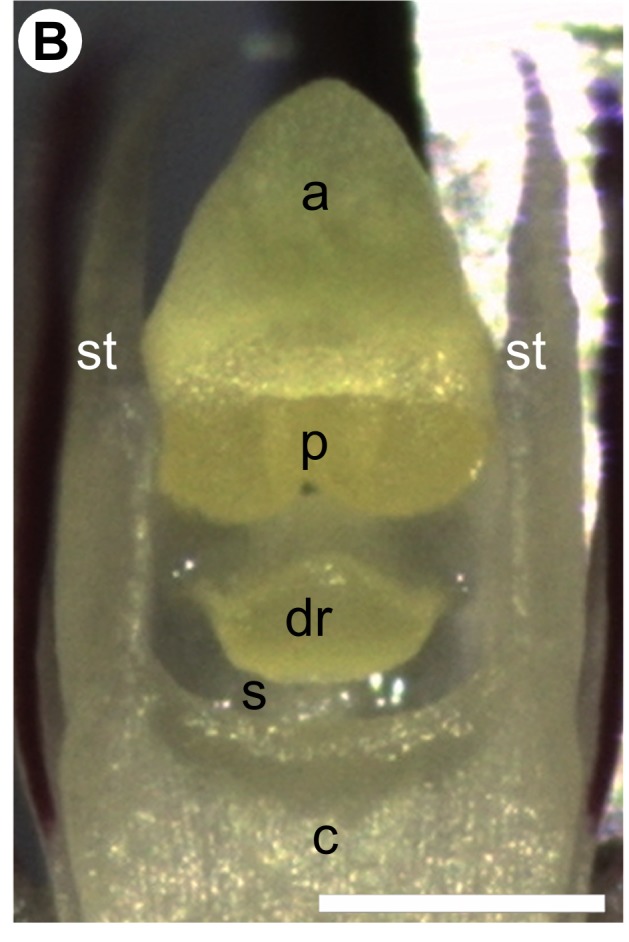# Correction: Histological and Micro-CT Evidence of Stigmatic Rostellum Receptivity Promoting Auto-Pollination in the Madagascan Orchid *Bulbophyllum* *bicoloratum*

**DOI:** 10.1371/annotation/447c32a9-0723-4fe9-bceb-3e24e0ef1cfc

**Published:** 2013-11-07

**Authors:** Alexander Gamisch, Yannick M. Staedler, Jürg Schönenberger, Gunter A. Fischer, Hans Peter Comes

The email address of the Corresponding Author is incorrect. The correct email address is: alexandergamisch@gmx.at.

In addition, there was an error in Figure 2B. A corrected version of Figure 2B can be viewed here: 

**Figure:**